# How can social needs impact on meaningful sports consumption?

**DOI:** 10.3389/fpsyg.2022.1043080

**Published:** 2022-11-03

**Authors:** Wang Zhigang, Guo Kai, Wang Chao, Duan Hongyan, Zhang Lei, Xue Zhao

**Affiliations:** ^1^Department of Economy and Management, Wuhan Sports University, Wuhan, China; ^2^Hengdian College of Film and Television, Jinhua, China

**Keywords:** meaningful sports consumption, social needs, team affiliation, self-improvement, self-esteem

## Abstract

The main goal of this study is to explore the drivers of meaningful sport consumption and its influence mechanism. In sports consumption, consumers not only seek hedonic value but also pursue to experience greater purpose and meaning in life, which is regarded as meaningful sports consumption. This study extends existing sports management literature by examining how social needs impact meaningful sports behavior with team affiliation, self-improvement, and self-esteem as mediators. Based on the questionnaire data collected from China, the empirical analysis results show that social needs have a significant positive impact on meaningful sports consumption behavior through the mediating effect of team affiliation and self-esteem motivation. However, self-improvement motivation does not have a mediating effect on the relationship between social needs and meaningful sports consumption. This study enriches the research content of sports consumption, adds research object of social needs, and expands the research scope of meaningful consumption by introducing meaningful sports consumption into the above domain.

## Introduction

Nowadays, people participate in sport activities greatly in both developed and emerging countries, forming a huge growing sports consumption market (Andreff and Andreff, 2009). Sports consumption means the expenditure of consumer spend on sports, which includes the consumption in all kind of sports products or services such as sportswear and equipment, watching sports games, expense in sports media, expense in sport participation, and etc. (Lera-Lopez and Rapun-Garate, 2007). Because the sports industry is becoming a growing economic sector, a large number of companies provide a wide range of sport products and service to this market (Andreff and Andreff, 2009). As an important social and economic phenomenon, sports consumption attract the attention of a lot of research (Karg and McDonald, 2011; Thibaut et al., 2014). These research involve determining factors of sports consumption (Thibaut et al., 2014), motivation of sports consumption (Byon et al., 2020), influence of sports consumption (Oh et al., 2022), and so on.

Previous studies on sports consumption focus on hedonic sports consumption, which means consumers pursue hedonic benefits from the consumption (Hall, 2015). However, besides pure pleasure, consumers also experience deeper meaning and greater purpose in life, which is conceptualized as meaningful sports consumption by researchers (Jang et al., 2020). For example, audience appreciate athlete’s moral beauty of helping competitors or others in competition, which is regarded as meaningful behavior, for it will be more prosocial in the future (Jang et al., 2019). Therefore, consumers want to find deeper meanings from human greatness and moral excellence as well as simply positive affects in their lives in sports consumption (Wirth et al., 2012).

Although meaningful sports consumption has been proposed to represent a unique behavior of sports consumption to distinguish traditional hedonic sports consumption, the studies on the area are very rare. Jang et al. (2019) suggested that meaningful messages can improve sports consumers’ supportive behavior of athletes’ foundation (Jang et al., 2019). In addition, Jang et al. (2020) discussed how will consumer response to meaningful sports consumption psychologically and behaviorally in different meaningful sports consumption context. However, what trigger consumers’ meaningful sports consumption and the mediating mechanism are not explored. Therefore the main goal of this study is to explore the drivers of meaningful sport consumption and its influence mechanism.

Individuals’ need is the drive of their consumption behavior. Compared with hedonic consumption, meaningful consumption usually arise from social needs (Syrjälä et al., 2015). Meanwhile, individuals’ need is usually the antecedent of their motivation of behavior, including consumption behavior (Taljaard and Sonnenberg, 2019). For individuals, team affiliation, self-improvement, and self-esteem are motivations arisen from social needs but not from hedonic need (Jang et al., 2020). Therefore, this study constructs a theoretical model based on the theory of hierarchy of needs and sports consumption motivation in a meaningful sports consumption context, with social needs as the antecedent variable and team affiliation, self-improvement, and self-esteem consumption motivation as the mediator. The sports consumption in this study refers to the universal consumption behavior in sports, including watching sports game, purchase of sportswear, participation of sports. The study explores forming mechanism of meaningful sports consumption, deepening the research on meaningful sports consumption. At the same time, it provides suggestions for sports companies to enhance the values of their products or services by endowing more humanistic significance to products or service.

### Literature review

#### Meaningful sports consumption

Meaningful sports consumption can be divided into two types based on different ways of self-construal. One is from a self-oriented perspective that highlights the extraordinary skills of athletes that make sports consumption meaningful, and the other is from an other-oriented viewpoint that emphasizes the exceptional moral qualities of athletes that make sports consumption meaningful (Jang et al., 2021). In a study on sports media consumption, it is found that audience have unique emotional dispositions toward athletes or teams. Audience’s positive emotions are enhanced when teams or athletes with positive tendencies win or when teams or athletes with negative spirits lose (Chiu and Won, 2022). However, in actual sports events, inconsistencies between game results and spectators’ expectation can lead to negative spectator emotions. Therefore, to attract audience to watch the game, sports media may bring them alternative satisfaction by evoking a meaningful feeling (Hall, 2015). For example, the sense of connection established between the audience and the team, the player, or other fans can awaken the individual’s moral perception of team spirit and team loyalty (Winegard and Deaner, 2010), thus increasing the game’s attractiveness. Sports media consumption can stimulate meaningful social cognitive experiences that foster human insight, significant perception, and socially connected emotional well-being in a more complex and sustainable way (Oliver and Bartsch, 2010; Oliver and Hartmann, 2010; Wirth et al., 2012). Rogers (2018) likewise points out that sports media can provide both pleasurable enjoyment and meaningful experiences for viewers, suggesting that sports consumption can be a more profound, significant consumption experience (Rogers, 2018). Sports consumers also view sports media entertainment that showcases athletes’ exceptional skills (Biscaia et al., 2012), their spirit in overcoming obstacles to reach their goals (Onu et al., 2016), and inspirational stories (Oliver et al., 2018) as meaningful consumer experiences.

The early sports management literature focused on how participation in various sports consumption provides hedonic benefits to sports consumers and determines their subsequent consumption behavior (Biscaia et al., 2012). As a new conceptualization of consumption, the most intuitive basis for distinguishing between hedonic and meaningful sports consumption is the triggering of self-transcendent emotions, namely the “higher” intrinsic needs identified in self-determination theory. Specifically, participation in meaningful sports consumption leads to a greater sense of upliftment than participation in hedonic sports consumption, as it provides opportunities for personal growth and self-development and is a critical component of self-transcendence (Jang et al., 2020).

While previous research has found that positive affect is a significant predictor of entertainment (David et al., 2008), a finding that is consistent with popular perception, Rogers (2018) also found a significant positive correlation between negative affect and entertainment. Oliver discusses why the public goes for tear-jerking, sad films (Oliver, 1993). The entertainment experience in such movies may not be hedonistic but rather provide viewers with a sense of meaningful connection, insight into the human condition, and the opportunity to explore complex moral issues (Tamborini, 2011). The motivation for watching sad movies is no longer the entertainment motivation of seeking a single dimension of pleasure but the happiness motivation of pursuing an additional size of meaning in life (Oliver, 2009). Oliver further confirmed in his study that happiness motivation is an antecedent variable that influences meaningful consumption behaviors such as watching a tragedy or witnessing moral beauty in the audience (Oliver and Raney, 2011).

By introducing the sense of self-improvement variable in study, Jang et al. (2019) confirmed that people trigger a sense of individual improvement by watching meaningful videos, enhancing their intention further to share these videos as a form of pro-social behavior (Jang et al., 2019). Bartsch et al. (2018) explored how empathy induced by the image of Paralympic athletes can indirectly influence public awareness and destigmatize through the negative emotion of pity to lead to a general shift in pro-social attitudes toward people with disabilities among sports viewers.

In summary, meaningful sports consumption is not purely hedonic or associated only with positive emotional experiences. It also provides some emotional experiences that can be interpreted as unfavorable, resulting in feelings of meaningfulness. In other words, meaningful sports consumption seeks not only to experience positive emotions but also to be driven by happiness and motivation to pursue deeper life goals and meaning (Rieger and Hofer, 2017).

#### Theory of sports consumption motivation

Motivation is defined as “the behavior of people that creates an internal drive to move toward a desired goal” (Armstrong et al., 2014). And consumer motivation is the consumer’s perceived demand function through the consumer decision process to become the driving factor, catalyzing the formation of willingness to buy or achieve purchase behavior (Iso-Ahola et al., 1999). Based on the definitions of motivation and consumer motivation, scholars have conceptualized the theory of sports consumption motivation from sociological and psychological perspectives.

There are different ways to classify the dimensions of sports consumption motivation in current studies. Wann was the first to develop the Sports Consumption Motivation Measure Scale (SFMC) using the sociology of sport theory, which classified sports consumption motivation into eight dimensions, including mild stress, self-esteem, escape, recreation, economy, esthetics, group belonging, family needs and so on (Wann, 1995). Milne and McDonald further proposed the Motivations of Sport Consumer (MSC) theory, which includes 12 motivational factors, including self-esteem, self-actualization, social interaction, and a sense of affiliation (Milne and McDonald, 1999). Funk et al. (2009) considered the shortcomings of existing research results in terms of complex dimensions and unfavorable application practices and optimized sports consumption motivation into five dimensions, namely socialization, entertainment, excitement, self-esteem, and distinction, developing the SPEED scale accordingly.

Different consumption motives will cause different consumption behaviors. Therefore, different dimensions of sports consumption motives will develop different nature of sports consumption behaviors accordingly. For example, in conspicuous consumption research, it has been demonstrated that self-esteem motivation can significantly influence apparent consumption behavior (Thoumrungroje, 2014). Chen explored the relationship between the motivation of professional soccer club fans to watch matches and their purchasing behavior in terms of the dimensions of star-following motivation, hobbies, and motivation to gain a sense of regional affiliation (Chen et al., 2014). Chu et al. (2019) examined the impacts of team affiliation, self-improvement motivation, and consumer engagement on Chinese travelers’ electronic word-of-mouth.

In summary, team affiliation, self-improvement, and self-esteem motivations are essential motivations of consumers, which essentially reflect consumers’ pursuit of meaningful values beyond the intrinsic use and enjoyment values of material goods. Therefore, this paper selects team affiliation, self-improvement, and self-esteem motivations as the focus of the study in the context of meaningful sports consumption to explore how these three types of sports consumption motivations play a mediating role in the relationship between social needs and significant sports consumption.

#### Social needs

The need is a subjective consciousness that arises when the organism lacks a particular substance and is the internal reaction of the organism to objective things. Unlike the single natural biological needs of animals, human needs also include social needs (Gong and Zhao, 2013). Social needs is a particular need acquired by individuals through various experiences during the growth process, which is a kind of advanced human need that includes love, affection, affiliation, and acceptance (Li et al., 2021). As a social being, an individual’s natural physical needs have become socialized into personal needs in the process of socialization. At the same time, some social needs beyond the natural ones are generated (Wang, 2003; Rodrigues et al., 2018). Man is a social being and has a deep need to share, help, and feel part of a group (Fromm, 1955). According to Maslow’s hierarchy of needs theory, “physiological needs” and “security needs” can be classified as natural human needs. In contrast, “social needs, “respect needs,” and “self-actualization needs” are classified as social needs (Yu, 1992). As to people in real life, when a certain kind of need is satisfied, they will further pursue a need at a higher level. The pursuit of higher level needs, the meaning of life, and a better life will become the internal motivation that drives their behavior (Maslow and Xu, 2007).

Social needs have been studied in a variety of fields. Steverink and Lindenberg (2006) investigated how the satisfaction of three human social needs, including affect, behavioral validation and social status, was associated with age, physical loss, and subjective well-being (Steverink and Lindenberg, 2006). It concluded that behavioral validation needs were more difficult to satisfy at higher levels of physical loss. However, none of the three social conditions became less important with the growth of age. Suki (2013) used multiple regression analysis to verify that the need for sociability significantly influenced students’ reliance on smartphones. Yu (1992) discussed the decisive role of social conditions on the development of library business. From the perspective of resource providers, Kong and Zhao (2018) verified that social demand significantly influenced the willingness and behavior of resource providers to participate in sharing economy platforms. Cao et al. (2013) demonstrated that two kinks of social needs, namely emotional belonging and social presence, would jointly positively impact satisfaction with social networking services and intention to continue participation. For social needs is an important drive of consumers’ behavior, particularly for the pursuit of values or meaning of life, this paper sets social needs as an antecedent variable to explore whether social needs positively influences meaningful sports consumption behavior.

### Model

The study introduced social needs as the independent variable, and use the motivation of team affiliation, self-improvement, and self-esteem in sports consumption as mediating variables. Meaningful sports consumption is the dependent variable in the study to explore the mechanisms of how social needs influence sports consumption behavior. Sports consumption motivation is the direct cause of people’s sports consumption behavior. Different sports consumption is driven by different sports consumption motivation. Yet, needs are the subjective desire state of consumers due to the lack of certain things (Chen et al., 2010). The satisfaction of lower-level needs reflects more hedonic considerations. In contrast, the pleasure of higher-level needs (such as social needs) reflects more concern for the happiness and the meaning of life (Oliver and Raney, 2011). Consumer groups with social needs often have a strong sense of family and social subordination, so they regard themselves and the country and society as an inseparable whole and pursue the emotional experience of affiliation to a team in the process of sports consumption (Zhang, 2002). Self-improvement motivation is significantly associated with individual pro-social behavior (Seo and Scammon, 2014), which helps individuals to establish a good impression in social interactions, gain positive recognition from others (Chu et al., 2019) and promote consumer engagement. Self-esteem is the motivation of individuals and groups to achieve higher spiritual and material needs (Funk et al., 2016). The strength of self-esteem motivation significantly affects consumers’ decisions and behaviors (Grubb and Grathwohl, 1967). Accordingly, this paper constructs a conceptual model in which social needs influence the formation of meaningful sports consumption behaviors, which is mediated by team affiliation, self-improvement, and self-esteem.

### Hypothesis

#### The impact of social needs on meaningful sports consumption

**Figure 1:**
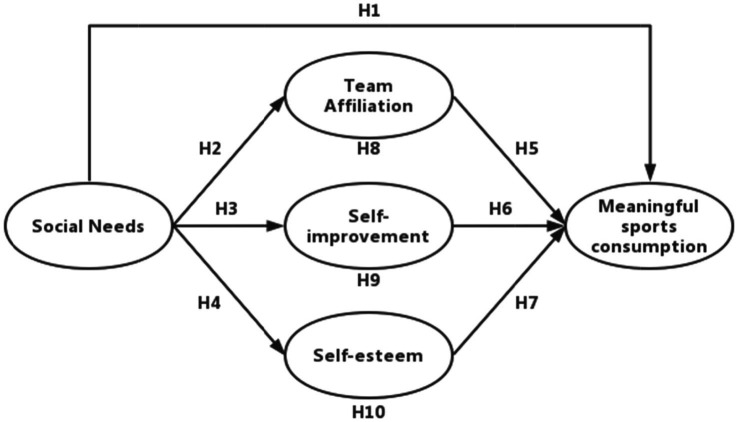
Framework of model.

The intrinsic need is the primary driver of all consumer behavior, thus we can predict consumer behavior with their needs (Yin, 2007). As to fitness consumption, Shi (2021) classify types of demand orientations into hedonic, physical, and social needs. Social needs reflect individuals’ higher-level needs, which will give rise to higher-level value demands, significantly influencing consumers’ fitness consumption behavior (Shi, 2021). In a consumer-led economy, sports products is evolved to meet the needs of consumers (Zhang, 2005). Wang and Li (2021) pointed out that the need for social recognition is a critical factor in the consumption of green products, which not only have functional ecological benefits (Sadovnikova and Pujari, 2017), but also trigger social and moral values in the consumption process (Muralidharan and Xue, 2016). Higher levels of social needs influence consumers’ generation of meaningful sports consumption behaviors. If consumers’ social needs are higher, then consumers will engage in relatively higher levels of significant sports consumption behaviors. Accordingly, the following hypotheses are proposed in this study (Figure 1).

*H1:* Social needs significantly and positively influences meaningful sports consumption behavior.

#### The impact of social needs on motivation of team affiliation

People’s intrinsic need for sports drives sports consumption motivation, that is the need for sports consumption is the basis for the forming of sports consumption motivation (Liu, 2000). Individuals’ sports consumption needs are different, making their consumption motives and behaviors different. Among sports consumption, although physical fitness is the most basic kind of sports needs that most people pursue, social needs, which focus on seeking collective identity and realizing self-worth, is also an considerable pursuit of sports consumers. This feeling of affiliation to other individuals or social groups is an instinctive human psychological need (Baumeister and Leary, 1995). Zhang (2002) pointed out that viewers who pursued collective identity-seeking will show a solid motivation to team affiliation to sports teams, which represented a certain group, region, ethnicity, or country. When major competitions involving national interests and prestige are concerned, they are more likely to give rise to their sense of home and national consciousness (Zhang, 2002; Kolesovs, 2021). Accordingly, the social needs is supposed to give rise to the consumption motive of team affiliation. Therefore, this paper proposes the following hypothesis.

*H2:* Social needs significantly and positively influences consumers’ motivation of team affiliation.

#### The impact of social needs on motivation of self-improvement

Self-improvement motivation refers to the desire of individuals to enhance their self-concept and acquire a good self-image (De Angelis et al., 2012). In meaningful sports consumption situations, self-improvement-oriented individuals are more inclined to receive feedback that can enhance the spiritual aspects of their personality, morality, and social values in the future. Seo and Scammon (2014) demonstrated a significant positive correlation between interdependence among social groups and self-improvement motivation in the pro-social behavior of helping people. In interpersonal interactions, the satisfaction of interdependence reflects the pursuit of people’s need for sociality. Leary (2007) argued that self-improvement motivation is rooted partly in people’s concern for social approval and acceptance that can protect people’s pursuit of subjective social well-being (Leary, 2007). Therefore, it can be argued that the most crucial function of self-improvement motivation is to satisfy social needs such as social interaction and interpersonal communication. Accordingly, this paper proposes the following research hypothesis.

*H3:* Social needs significantly and positively influences consumers’ motivation of self-improvement.

#### The impact of social needs on the motivation of self-esteem

Currently, the theories related to self-esteem include dominance theory, social scale theory, and fear control theory, all of which jointly point out that self-esteem is an individual’s self-evaluation of their social attributes, that is, self-esteem refers to an individual’s subjective evaluation of their self-value as a human being (Orth and Robins, 2014). Funk et al. (2016) found that self-esteem, respect, and ambition are the key motives associated with the satisfaction of self-achievement needs in the consumption process. Once individuals wish to achieve more social recognition than others, they will not only have a stronger sense of self-consciousness but will also inspire higher levels of self-esteem. According to Maslow’s hierarchy of needs theory, the highest level of social needs is the need for self-actualization. In addition, in the organism theory, it is stated that one of the leading human needs tendencies to reach self-actualization is the need for self-improvement (Ford, 1991). Meanwhile, Baumeister (1997) pointed out in his study that the need for self-improvement is an innate drive for individuals to improve their self-esteem (Baumeister, 1997). In summary, social needs influences self-improvement motivation through its self-actualization needs dimension, which further influences individuals’ self-esteem motivation through self-improvement motivation. Accordingly, the following hypothesis is proposed in this paper.

*H4:* The consumers’ social needs significantly and positively influences their self-esteem motivation.

#### The impact of team affiliation motivation on meaningful sports consumption behavior

Ko et al. (2017) pointed out that sports consumers actively seek opportunities to satisfy their motivation to affiliation to a team by participating in sports clubs or watching sports teams play and create a sense of affiliation by engaging in various sports experiences. Related studies took team affiliation motivation as one of the essential dimensions influencing fans’ motivation to watch games and concluded that spectators with strong team affiliation motivation generally have strong ethnic sentiments (Mutz and Gerke, 2018). When it comes to their own ethnic team’s games, they are also the most emotionally invested in the fun. This kind of emotion tend to dominate the atmosphere and emotional reactions in the games. Funk et al. (2001) found that team-attached consumers take pride in their community and watch more community-related games on television (Funk et al., 2001), while the international nature of World Cup events replaces this community pride with a corresponding sense of national pride. The consumption phenomenon of watching games supporting one’s team out of ethnicity or regional affiliation can be categorized as meaningful sports consumption behavior. With this in mind, the following hypotheses are proposed in this paper.

*H5:* consumers’ team affiliation motivation significantly and positively influences meaningful sports consumption behavior.

#### The impact of self-improvement motivation on meaningful sports consumption behavior

Material wealth is essential to the concept of self and can even be seen as an extension of the self, thus images related to the self (e.g., self-improvement, self-esteem, self-consistency) influence consumers’ purchases of material goods (Lu, 2019). In the context of research on sports consumption, Jang et al. (2020) found that sports consumers are more likely to support athlete-run charitable foundations through a sense of self-improvement when viewers watch promotional videos that show the meaningful behavior of athletes. At the same time, sports events or promotional videos that showcase athletes’ outstanding ethics or superior skills can lead to a greater sense of uplift among sports consumers, which significantly determines the consequences of their behavior (Jang et al., 2019). Emotional triggers for self-transcendence (e.g., elevation, admiration, and awe) have also emerged as critical factors in helping scholars conceptualize meaningful consumption (Oliver et al., 2018). Accordingly, the following hypotheses were formulated in this study.

*H6:* Self-improvement motivation significantly and positively influences meaningful sports consumption behavior.

#### The impact of self-esteem motivation on meaningful sports consumption behavior

Self-esteem motivation can impact how people behave and has a motivating effect on some of their behaviors, which is one of the main drivers that motivate consumer decisions and behaviors (Grubb and Grathwohl, 1967; Philp et al., 2018). It is indicated that from the perspective of status consumption self-esteem motivation can significantly and positively promote consumers to consume certain social status goods that can represent their attributes or status expectations (Liu and Li, 2022). Products related to status have not only simple use value, but also have symbolic added value (McDonald et al., 2002). Meanwhile, studies on how self-esteem motivation influence consumer behavior have also focused on the area of conspicuous consumption (Khan and Dhar, 2006; Widjajanta et al., 2018), in which a significant positive relationship between self-esteem motivation and conspicuous consumption has been confirmed. In essence, conspicuous consumption is a kind of consumer behavior in which people seek a higher level of spiritual satisfaction and social identity after satisfying their basic material needs. The process is also given the symbolic meaning of displaying oneself, which has the same connotation as meaningful consumption. The nature of self-esteem motivation influencing consumption behavior also applies to the meaningful sports consumption context, where individuals with higher levels of self-esteem motivation tend to consume the significant value behind sports. Accordingly, this paper proposes the following hypothesis.

*H7:* Self-esteem motivation significantly and positively influences meaningful sports consumption behavior.

#### The mediating role of team affiliation

Consumer behavior is determined by the diverse needs of consumers. Namely different needs will induce different consumption motives. And then, based on the nature of the dominant motive among the many consumption motives, corresponding consumption behavior is induced. Wann et al. (2001) suggested that sports event spectators can motivate team affiliation by sharing social identities with other fans or social groups (Wann et al., 2001). Shared social identities reflect the social interaction dimension of the public’s social needs, which generate the motivation to affiliation to a team. Feeling connected and socially interacting with each other is a social experience of watching sporting events. When social needs are met through belonging experiences, a sense of well-being and satisfaction is generated accordingly (La Guardia et al., 2000). Chu et al. (2019) explored the relationship between affiliation motivation and consumer engagement using Chinese consumers’ WeChat friend circle as a research object. The study showed that Chinese consumers’ higher sense of affiliation or attachment to their friend circle positively influences their WeChat consumption engagement. In summary, social needs motivate consumers’ motivation to affiliation to a team, and this motivation further affects consumption behavior. Accordingly, this paper proposes the following research hypothesis.

*H8:* Team affiliation motivation plays a mediating role in the relationship between social needs and meaningful sport consumption behavior.

#### The mediating role of self-improvement

Individuals need to meet the need from group or society and meet the personalized need, such as the realization of self-value, which determines the behavior of personal sports consumption tendency and consumption motivation. And self-improvement motivation is an important mechanism that mediates the influence of the external social environment on the internal self, which can help people innovate their self-behavior, form a long-lasting and stable self-system, and become a significant motivation to mark the self (Kurman, 2006). It has also been shown that individuals will continuously improve themselves to achieve the behavioral standards assigned by specific social roles to become a better self in a given society, which also suggests that the satisfaction of social needs within the broader framework of the social environment will further enhance individuals’ motivation for self-improvement. In the consumption context, it was found that when individuals are in a crowded social environment, they are more likely to be motivated by self-improvement and thus more inclined to engage in self-improvement consumption (Ding and Zhong, 2020). Consumption behaviors that enable individuals to perform a task better or enhance certain aspects of themselves are categorized as self-improvement consumption (Allard and White, 2015). In summary, self-improvement motivation is generated by the social needs of individuals. Under the improvement of this motivation, people are more likely to pursue those consumptions that can lead to self-improvement. Accordingly, this paper proposes the following research hypothesis.

*H9:* Self-improvement motivation has a mediating role in relationship between social needs and meaningful sport consumption behavior.

#### The mediating role of self-esteem

Self-esteem motivation reflects the extent to which a person believes that participation in sporting events provides an opportunity for alternative achievement (Funk et al., 2009). This opportunity for achievement and challenge gives individuals with individual and collective self-esteem an incentive to pursue new sports consumption experiences. Research has shown that when faced with the experience of success or failure of the team they support, such incidents are more likely to positively or negatively affect the satisfaction of personal well-being needs when individuals have higher levels of self-esteem (Kim et al., 2017). The satisfaction of happiness needs is one of the prerequisites of social needs to realize our pursuit of a good life (Schippers and Ziegler, 2019). It was noted that as consumers build and maintain self-esteem through association with sports teams, they are more likely to attend future games and purchase merchandize (Trail et al., 2005). Even in unsuccessful seasons, the adverse effects that affect continued participation are temporary for fans with high self-esteem (Bizman and Yinon, 2002). The nature of this consumption phenomenon is consistent with the connotations of meaningful sports consumption that we have explored. The strength of the social needs positively influences the level of self-esteem motivation of the individual. Meanwhile, the higher the level of self-esteem motivation, the stronger the connection between the individual and their favorite sports team or regional representative team, which in turn influences consumers’ subsequent consumption decisions and consumption behavior. Accordingly, this study proposes the following hypothesis.

*H10:* Self-esteem has a mediating role in the relationship between social needs and sport consumption behavior.

## Methodology

### Sample selection

In this paper, Chinese consumers who have participated in sports consumption were used as the respondents of a questionnaire survey to explore the influencing factors that affect consumers’ behavior of engaging in meaningful sports consumption. The subject of sports consumption refers to the ordinary individuals who buy sport product and service as consumers. The survey process was divided into a pre-survey and a formal survey. The questionnaire was reworked to address the pre-survey’s vague expressions and linguistic ambiguities. In the survey process, a combination of online questionnaire survey and offline distribution of paper questionnaire survey was used, and 355 questionnaires were distributed. 298 valid questionnaires were collected after eliminating invalid questionnaires such as too fast response time and regular distribution of answers. The descriptive statistics of sample is shown in the following Table 1.

**Table 1:** Descriptive statistics of samples.

Classification indicator	Categories	Frequency	Percentage (%)
Gender	Male	135	45.3
	Female	163	54.7
Ages	<18	96	32.2
	18–25	172	57.7
	26–35	21	7.0
	36–45	2	0.7
	>45	7	2.3
Education level	High school and below	58	19.5
	Junior college	71	23.8
	Undergraduate	96	32.2
	Master and above	73	24.5
Monthly income	<3,000	211	70.8
	3,000–5,000	41	13.8
	5,000–8,000	29	9.7
	>8,000	17	5.7
Monthly sports consumption expenditure	<200	181	60.7
	200–500	66	22.1
	500–1,000	33	11.1
	>1,000	18	6.0

### Measurement

The measurement scales for variables in this study is well established in existing studies. We use them as the measurement in this study with some items adjusted according to the actual situation of the study context. The process of scale development is as follow. Scales of variables in this study were collected from existing literature. Then the question items were adjusted according to the context of the study through group discussions. Finally, the scales of variables in this study are determined (see Table 2). This study includes the scales of social needs, team affiliation, self-improvement, self-esteem and meaningful sports consumption. The scale questions were based on the Likert 7-point scale, with 1 indicating complete disagreement and seven indicating entire agreement. As to the measurement questions, social needs refers to the study of Shen and Hu (2009). Team affiliation motives refer to the study by Zhang et al. (2016). Self-improvement motivation refers to the study of Jang et al. (2020). Self-esteem motivation refers to the study of McDonald et al. (2002). Meaningful sports consumption refers to the study of Jang et al. (2021). For the details of variables measurement, it can be seen in Table 2.

**Table 2:** Questionnaire items.

Latent variable	Item	Load	SD	AVE	CR	Cronbach’s *α*
Team affiliation	Sense of nationality and patriotic feelings	0.702	0.084	0.544	0.781	0.749
	Feel proud	0.777				
	Interest in relevant information	0.731				
Self-esteem	Self-esteem	0.900	0.114	0.813	0.928	0.911
	Self-confidence	0.949				
	Pride	0.854				
Self-improvement	Admire excellent skills	0.737	0.071	0.742	0.896	0.767
	Moral improvement	0.838				
	Enhance perseverance	0.702				
Meaningful sports consumption	Support domestic sports products	0.743	0.068	0.579	0.804	0.772
	Watch my country’s (local) team play	0.834				
	Buy sports tickets to support the development of domestic (local) sports	0.701				
Social needs	Healthy image	0.863	0.147	0.742	0.896	0.859
	Image of love for life	0.898				
	Social status and positive image	0.822				

## Results

### Measurement model test

Confirmatory factor analysis is used to test the reliability of the data, which shows that the measurement models fit well: *χ*^2^/df = 1.545, RMR = 0.137, GFI = 0.924, AGFI = 0.878, PGFI = 0.577, TLI = 0.897, CFI = 0.926, and RMSEA = 0.043. As shown in Tables 2, 3, combined reliability of all structural variables is above the recommended level of 0.70 and the average variance extracted (AVE) is also above the recommended level of 0.50, which indicates that this study has good reliability for the measurement of the structural variables in the study. The standardized factor loadings for all structural variables are higher than 0.7 and significant at the *α* = 0.01, indicating that the scale has high convergent validity. In addition, the square root of all AVEs is greater than their row and column correlation coefficients, which indicates that the scale has high discriminant validity.

**Table 3:** Correlation coefficient of latent variables.

	Social needs	Meaningful sports consumption	Self-improvement	Self-esteem	Team affiliation
Social needs	0.861				
Meaningful sports consumption	0.542	0.76			
Self-improvement	0.405	0.408	0.861		
Self-esteem	0.609	0.461	0.566	0.901	
Team affiliation	0.327	0.556	0.588	0.618	0.736

The diagonal data of the matrix represent the square root of the AVE values and the lower half of the matrix represents the correlation coefficient.

### Structural model test

**Figure 2:**
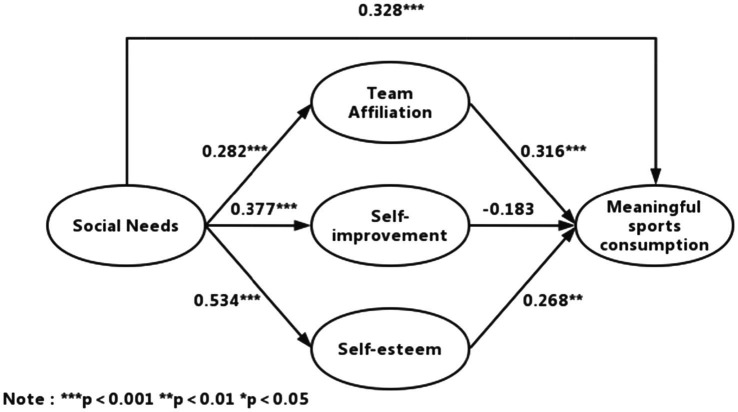
Path coefficient of conceptual model.

The model fit goodness-of-fit statistics are *χ*^2^/df = 1.841, RMR = 0.096, GFI = 0.949, AGFI = 0.910, PGFI = 0.538, TLI = 0.958, CFI = 0.973, and RMSEA = 0.053. These statistical values indicate that the structural model fits well. Figure 2 shows the results of the path analysis of the structural equation model.

The tests of the structural model included the estimated path coefficients and the tests of the model’s explanatory power. The model’s standardized path coefficients are shown in Figure 2. The social needs had a significant impact on both consumption motivation and meaningful sports consumption behavior, with meaningful sports consumption (*γ* = 0.328, *p* < 0.001), team affiliation (*γ* = 0.28, *p* < 0.001), self-improvement (*γ* = 0.377, *p* < 0.001), and self-esteem (*γ* = 0.534, *p* < 0.001). Consumption motivation had a partial effect on meaningful sports consumption, with team affiliation (*γ* = 0.316, *p* < 0.001), self-esteem (*γ* = 0.268, *p* < 0.01) having a significant impact on meaningful sports consumption, but self-improvement (*γ* = −0.183, *p* > 0.05) doing not have a significant impact. Therefore, research hypotheses H1–H7 are supported except for H6. There is no significant influence relationship between self-improvement motivation and meaningful sport consumption behavior, so there is no mediating effect of self-improvement motivation in the mechanism of the role of social needs to influence meaningful sport consumption, resulting in H9 failed to be supported.

### Mediating effect test

To verify the mediating effect of team affiliation and self-esteem, this paper uses the Boot-strap method to test with AMOS software. Using the Bias-corrected and Percentile method with 5,000 replicate samples, if the upper and lower bounds do not include zero values in the 95% confidence interval, it indicates that there exists significant mediating effect.

### Test for mediating effects of team affiliation

The mediating effect of team affiliation between social needs and meaningful sports consumption behavior is significant, with an indirect effect of 0.036, excluding 0 (0.009, 0.086) at 95% confidence interval and value of *p* < 0.05. Meanwhile, the direct effect of social needs on meaningful sports consumption behavior is significant, with a direct effect of 0.269, excluding 0 (0.175, 0.375) at confidence interval. It indicates a significant partial mediating effect of team affiliation in the influence of social needs on meaningful sports consumption (Table 4). Therefore, hypothesis H8 is supported.

**Table 4:** Test results of mediating effect of team affiliation.

Paths	Effect value	Boot SE	*p*	Bias-corrected 95% CI	
				Lower	Upper
Social Needs—Team affiliation—Meaningful sports consumption	0.036	0.018	0.003	0.009	0.086
Direct effect	0.269	0.050	0.000	0.175	0.375

### Test for mediating effects of self-esteem

The mediating effect of self-esteem between social needs and meaningful sports consumption behavior is significant, with an indirect effect of 0.073, excluding 0 (0.018, 0.145) at 95% confidence interval and value of *p* < 0.05. Meanwhile, the direct effect of social needs on meaningful sports consumption behavior is significant, with a direct effect of 0.234 and confidence interval not containing 0 (0.124, 0.342). it indicates a significant partial mediating effect of self-esteem in the influence of social needs on influence meaningful sports consumption therefore hypothesis H10 is supported (Table 5).

**Table 5:** Test results of mediating effect of self-esteem.

Paths	Effect value	Boot SE	*p*	Bias-corrected 95% CI	
				Lower	Upper
Social Needs—Self-esteem—Meaningful sports consumption	0.073	0.032	0.008	0.018	0.145
Direct effect	0.234	0.050	0.000	0.124	0.342

## Discussion

### Discussion of results

To explore the cause that drives consumers to conduct meaningful sports consumption behavior, this paper uses empirical analysis to discuss how the social needs of consumers influence their meaningful sports consumption through the mediating effect of team affiliation and self-esteem motivation. The discussion of the result is as follows.

#### Social needs and meaningful sports consumption behavior

It was indicated in studies that social needs of individuals can influence their behavior. For example, higher levels of social existence will encourage higher personal motivation to interact with others. The stronger the emotional affiliation in a virtual social network, the stronger the individual’s intention to use it (Cao et al., 2013). Madupu (2006) also pointed out that online sharing economy platforms have economic and social attributes, thus consumers can satisfy their social needs for belonging and identity by participating in virtual communities (Madupu, 2006). In the field of green consumption research, consumers’ social needs can significantly influence their attitudes toward energy-efficient products and their willingness to repurchase (Wang and Li, 2021). In the sports research field, encouraging social interaction in sports consumption can contribute to satisfying sociability needs by emphasizing a range of social values such as teamwork and peer camaraderie in sports (Kim and James, 2019). In this study, social needs also positively influence consumer behavior in sports consumption, which is consistent with the findings of previous studies. However, unlike previous studies, this paper further confirms that social needs can not only influence consumers’ general behavior but also influence consumers to take meaningful sports consumption behavior. This is reflected in the fact that individuals are increasingly concerned about the meaningful value behind sports consumption. Meaningful sports consumption provides consumers with an mix of experiences that satisfy their more diverse and deeper social needs.

#### Social needs and consumer team affiliation, self-improvement, and self-esteem motivation

Previous research has shown that market orientation is gradually shifting from providing customers with core products to providing higher consumer value, in which the value gained from the customer-enterprise partnership is an essential factor influencing customers’ sense of affiliation to the company (Liu, 2008). In studies of sports consumption, personality traits such as the arousal needs of sports spectators significantly affect individuals’ motivation to affiliation to a team (Donavan et al., 2005). This study also confirms the influence of social needs on team affiliation motivation in sports consumption context from the perspective of individual needs. Self-improvement motivation is an intrinsic drive spawned by consumers to satisfy the need for self-actualization in the social needs (Ford, 1991), which helps individuals to establish a good impression and gain positive recognition from others in social interactions (Chu et al., 2019). The same relationship between social needs and consumer’s self-improvement is confirmed again in this study. Unlike previous studies, this paper confirms that the social needs of sports consumers can significantly influence their self-improvement motivation in a new context, that is sports consumption. When individuals are in a consumption situation where they participate alone, the conflict between unmet social needs and the social attributes of experiential consumption can cause consumers to experience negative emotions such as loneliness, despair, and embarrassment, thus reducing their self-esteem (Li et al., 2021). Self-esteem, respect and ambition are key motivators in sports consumption associated with achievement need satisfaction (Funk et al., 2016). The relationship between social needs and self-esteem is also verified in this study. The difference in this study is that the relationship between social needs and self-esteem motivation also holds in the context of sports consumption. That is, the social needs becomes an antecedent variable affecting the level of consumer self-esteem motivation when sports consumption behavior is given more meaningful value.

#### Team affiliation, self-esteem motivation and meaningful sports consumption behavior

Previous research has shown that pride in the hometown team among fans from the same geographic region is an essential factor in initially identifying with a group. Pride in the hometown team reflects consumers’ affiliation attachment to their hometown, thus this motive of belonging can influence the propensity of sports fans to identify with the group they support (Ko et al., 2017). This sense of attachment to the team or national pride will encourage consumers to continue watching the game (Zhang et al., 2016; Widjajanta et al., 2018). This study further confirmed the sense of affiliation can induce consumers’ consumption behavior. Differently from previous studies, this paper further suggests that consumers with solid team affiliation motivation are more likely to engage in meaningful sports consumption behavior, besides common sports consumption.

Individuals with high self-esteem motivation see themselves as fully integrated with the team they support. The negative emotions associated with the team’s failure have a low impact on their willingness to continue participating in consumption (Bizman and Yinon, 2002). In other consumption domain studies, self-esteem motivation can significantly influence consumers’ conspicuous (Souiden et al., 2011; Widjajanta et al., 2018) and status (Liu and Li, 2022) consumption behaviors. Accordingly, consumers with high self-esteem motivation no longer seek positive emotional experiences or the practical and functional value of the goods they consume but rather a mix of emotional experiences or the symbolic and meaningful values behind the goods they consume. The finding of this study is consistent with previous research that individuals’ self-esteem can impact their consumption behavior. However, different from the previous research, this paper further suggests that the impact of sports consumers’ self-esteem motivation on consumption behavior also exists in the meaningful sports consumption domain.

#### Self-improvement motivation and meaningful sports consumption behavior

Study has confirmed that there is an association between self-improvement motivation and meaningful sports consumption behavior (Jang et al., 2021). However, this study confirms that in the context of Chinese sports consumption, self-improvement motivation does not significantly influence meaningful sports consumption behavior. The root cause of this phenomenon may lie in the unique traditional Chinese. Western countries are more interested in conquering nature, pursuing faster, higher, and firmer on the playing field, and showing the value of transcending oneself and nature. In contrast, traditional Chinese sports culture considers man and nature to be in a harmonious and symbiotic relationship, emphasizing on emotions and respecting moral concepts, which weakens sports’ competitive nature and emphasizes the value of righteousness over profit. The Western rational way of thinking is based on the spiritual connotation of competition and transcendence. This cultural concept of survival, improvement, and perfection in competition makes the more robust the motivation for self-improvement, the more meaningful sports consumption behavior can be generated. In contrast, the Chinese intuitive way of thinking is based on the value pursuit of “harmony” and “unity.” This cultural concept of sports, which emphasizes the cultivation of health and mind and weakens the competition for conquest, makes the self-improvement motive do not influence Chinese sports consumers to produce meaningful sports consumption behavior.

#### Mediating effects of team affiliation and self-esteem motivation

Social needs are a state of organismic deficiency that recurs periodically, which can be achieved through participation in meaningful activities (Nezlek, 2022). Meaningful sports consumption behavior can satisfy this particular need. In this satisfaction process, the individual’s motivation to achieve the team affiliation to the nation or territory and the pursuit of self-esteem motivation play a significant mediating role. When sports fans psychologically associate themselves with their local sports teams, they form an “imaginary intimate relationship” with the team, in which social values are experienced through this relationship (Greendorfer and Bruce, 1991). When this kind of sports fans cheer for the same team, the sense of affiliation experienced through various direct or indirect social contacts can positively impact the individual’s subjective well-being (Kim and James, 2019). In this sense, meaningful sports consumption experiences can satisfy team affiliation motivation. Self-esteem motivation arises from an individual’s self-concept that gradually develops during socialization, which can influences the initiative an individual takes when adapting to society (Sherbourne and Stewart, 1991). The self-esteem gains of such sports fans often come from a sense of identification with the team and a sense of affiliation, in which their own value will be reflected through the meaningful value in meaningful sports consumption.

Past research on conspicuous consumption has confirmed the partial mediating effect of self-esteem between economic status and conspicuous consumption tendencies (Yuan, 2011), in which social media use can increase individuals’ self-esteem levels and further enhance consumers’ tendency to engage in conspicuous consumption (Widjajanta et al., 2018). Team affiliation motivation has been studied in the domain of how users behave in virtual communities (Sutanto et al., 2011), where the social value perceived by users when participating in virtual communities positively influences community users’ sense of affiliation, in which the emotional attachment between users and the community further positively influences users’ willingness to participate (Zhao and Liu, 2018).

This study further confirms the mediating effects of self-esteem and team affiliation motives on consumption behavior in the context of meaningful sports consumption. It verifies the existence of partial mediating effects of self-esteem and team affiliation in the process of social needs to influence meaningful sports consumption behavior.

### Theoretical contributions

This paper focuses on the internal mechanism of meaningful sports consumption formation. This paper develops a theoretical model for meaningful sports consumption behavior from the perspective of social needs, verifying the mediating role of team affiliation and self-esteem motivation. This study explains the essential internal dynamics in forming meaningful sports consumption, further developing the research on meaningful sports consumption. The specific theoretical contributions are as follows.

First, this study enriches existing sports consumption research by discussing the issue of meaningful sports consumption. Although research on meaningful behavior has emerged in several disciplines, including psychology (Winegard and Deaner, 2010), journalism and media (Hall, 2015), and sociology (Bartsch et al., 2018), the concept of meaningful consumption has been grossly neglected in the field of sports management. Most sports consumption research has focused on the hedonic aspects of sport consumption (Biscaia et al., 2012), neglecting the component of meaningful sports consumption. The newly emerged research on meaningful sports consumption only discussed the impact of this behavior on consumers’ emotions, motivations, and behavioral outcomes (Jang et al., 2021). This study takes meaningful sports consumption as the objective of research, discussing the formation mechanism of “meaningful sport consumption behavior” from the perspective of consumer demand, finding consumers’ social needs can induce their meaningful sports consumption behavior, in which team affiliation and self-esteem motivation plays the mediating role. This study introduces new research objects into sports consumption behavior, further enriching the study in this field.

Second, this paper further enriches the research object of social needs by exploring the influence of social needs on meaningful sports consumption behavior in the field of sports consumption. Current research on social needs has focused on the fields, such as children’s cell phone addiction (Suki, 2013), green consumption (Wang and Li, 2021), public service development (Yu, 1992), and virtual community participation (Kong and Zhao, 2018). Most studies on social needs have only concerned the impact of social needs on general consumption behaviors, in which its impact on meaningful consumption behaviors was neglected. While this study explores the impact of social needs on meaningful consumption behavior, particularly meaningful sports consumption behavior, which enriches the study of social needs by introducing a new research object.

Finally, this paper further expands the research scope of meaningful consumption by stretch it into the field of sports consumption. Meaningful consumption has been discussed in ordinary consumption domain. For example, meaningful consumption is seen as a systematic symbolic manipulation behavior (Baudrillard, 1988), where people are keen on a commodity brand because of the pursuit of ideological values within the symbol, which may be symbolic meanings such as status, wealth, and taste. Meaningful consumption behavior has been effectively explored in the green product consumption filed, where the environmental attributes behind consumers’ consumption of energy-efficient products are given some moral significance (Wang and Li, 2021). In media consumption, people watching sad movies can experience more complex emotional reflections of bitterness, introspection, and compassion, reflecting a deeper insight into the human condition and a broader understanding of the meaning of life (Oliver and Raney, 2011). Meaningful sports consumption behavior is seldom discussed separately in the research on meaningful consumption. This paper enriches the research object of meaningful consumption by introduce meaningful sports consumption behavior into this field.

## Conclusion

This study examines the causes of meaningful sports consumption behavior among sports consumers from the perspective of consumer demand motivation and draws the following conclusions from the above findings.

First, as a higher level of need for individual growth and development, social needs can significantly and positively influence consumers to produce meaningful sports consumption behaviors. Second, social needs significantly affect consumer motivations for team affiliation, self-esteem, and self-improvement. Third, different consumption motives are intrinsic to the generation of different consumption behaviors. The generation of meaningful sports consumption behaviors by sports consumers is mainly positively influenced by individual team affiliation and self-esteem motives. At the same time, self-improvement motives that only emphasize individual development do not significantly influence consumers to produce meaningful sports consumption behaviors. Fourth, social needs can further influence consumers to produce meaningful sport consumption behaviors through the mediating role of team affiliation and self-esteem motivation.

## Practical recommendations

1. Companies in sports business should pay attention to consumers’ meaningful sports consumption behavior and strengthen this concept in sports consumption. The current consumption structure of residents has been upgraded from material consumption, actual consumption, and developmental consumption to comfort consumption, pleasure consumption, and health consumption. Sports enterprises should actively analyze the drive behind the phenomenon of meaningful sports consumption. For various sports consumption, such as fitness, watching games, buying sports equipment, etc., there is not only one kind of value for them. However, its original “meaning” is replaced by a new meaning according to the needs and desires of consumers after combining it with their daily lives. Therefore, the products or services enterprises provide should be based on consumers’ actual needs and motivations and provide consumers with more sports consumer goods to meet their value-seeking.
2. Companies in sports business should meet consumers’ social needs and improve the meaningful value of their goods. The phenomenon of meaningful sports consumption shows that it is hard to meet the comprehensive needs of sports consumers through sports goods for material survival or functional value. Therefore, sports enterprises should take the social needs of consumers into consideration and realize the enterprise’s long-term development by satisfying the varied emotional experience or meaningful value of consumers in the consumption process.
3. Companies in sports business should improve the team attribute value of sports goods to enhance consumer stickiness between customers and enterprises. Consumer team affiliation motivation is one of the critical motivations influencing consumers to engage in meaningful sports consumption behavior. Consumers with such motives tend to have a strong sense of national patriotism. Therefore, event operating companies should highlight more national and regional characteristics in the layout of venues and pre-game publicity to create an intense home country atmosphere.
4. Companies in sports business should strengthen self-esteem features to its products or service to increase consumers’ willingness to consume sustainably. When consumers establish and maintain self-esteem connections with sports teams, they are more likely to attend future games and purchase peripheral merchandize. Therefore, for event organizers they should foster this connection through pre-game and post-game activities participated by coaches, athletes, and fans. In addition, dedicated areas should be provided within the event venue for such loyal fans and provide more detailed services to enhance or sustain the level of consumer self-esteem. Moreover, the awakened self-esteem perception can be used to initiate season tickets and membership of the following season after the season ends.

## Data availability statement

The raw data supporting the conclusions of this article will be made available by the authors, without undue reservation.

## Author contributions

WZ: conceptualization. GK: draft writing. WC: data collection. DH: data analysis. ZL: project administration. XZ: resource provision. All authors contributed to the article and approved the submitted version.

## Funding

This research was funded by the Young and middle aged scientific research team of Wuhan Sports University in 2021 (21KT18), Philosophy and social science research project of Hubei Education Department (19Y099), Scientific research project of Hubei Education Department (B2021186), and Youth Scientific Research Fund of Wuhan Sports University (2022S05).

## Conflict of interest

The authors declare that the research was conducted in the absence of any commercial or financial relationships that could be construed as a potential conflict of interest.

## Publisher’s note

All claims expressed in this article are solely those of the authors and do not necessarily represent those of their affiliated organizations, or those of the publisher, the editors and the reviewers. Any product that may be evaluated in this article, or claim that may be made by its manufacturer, is not guaranteed or endorsed by the publisher.
